# Knockout of the two evolutionarily conserved peroxisomal 3-ketoacyl-CoA thiolases in *Arabidopsis* recapitulates the *abnormal inflorescence meristem 1* phenotype

**DOI:** 10.1093/jxb/eru397

**Published:** 2014-10-07

**Authors:** Andrew A. G. Wiszniewski, John D. Bussell, Rowena L. Long, Steven M. Smith

**Affiliations:** ^1^ARC Centre of Excellence in Plant Energy Biology, The University of Western Australia, 35 Stirling Highway, Crawley, WA 6009, Australia; ^2^Max-Planck Institute for Molecular Plant Physiology, Wissenschaftpark Golm, Am Mühlenberg 1, D-14476 Potsdam, Germany

**Keywords:** 3-Ketoacyl-CoA thiolase, *Arabidopsis thaliana*, β-oxidation, flowering, germination, peroxisome, seed development.

## Abstract

A specific function for peroxisomal β-oxidation in inflorescence development in *Arabidopsis thaliana* is suggested by the mutation of the *ABNORMAL INFLORESCENCE MERISTEM 1* gene, which encodes one of two peroxisomal multifunctional proteins. Therefore, it should be possible to identify other β-oxidation mutants that recapitulate the *aim1* phenotype. Three genes encode peroxisomal 3-ketoacyl-CoA thiolase (KAT) in *Arabidopsis*. *KAT2* and *KAT5* are present throughout angiosperms whereas *KAT1* is a Brassicaceae-specific duplication of *KAT2* expressed at low levels in *Arabidopsis*. *KAT2* plays a dominant role in all known aspects of peroxisomal β-oxidation, including that of fatty acids, pro-auxins, jasmonate precursor oxophytodienoic acid, and *trans*-cinnamic acid. The functions of *KAT1* and *KAT5* are unknown. Since KAT5 is conserved throughout vascular plants and expressed strongly in flowers, *kat2 kat5* double mutants were generated. These were slow growing, had abnormally branched inflorescences, and ectopic organ growth. They made viable pollen, but produced no seed indicating that infertility was due to defective gynaecium function. These phenotypes are strikingly similar to those of *aim1*. *KAT5* in the Brassicaceae encodes both cytosolic and peroxisomal proteins and *kat2 kat5* defects could be complemented by the re-introduction of peroxisomal (but not cytosolic) KAT5. It is concluded that peroxisomal KAT2 and KAT5 have partially redundant functions and operate downstream of AIM1 to provide β-oxidation functions essential for inflorescence development and fertility.

## Summary

Simultaneous mutation of two peroxisomal thiolase enzymes shows that fatty acid β-oxidation is required for the normal development of inflorescences in *Arabidopsis* and for successful fertilization to produce seed.

## Introduction

Peroxisomes of higher plants display plasticity of function depending on plant growth stage and tissue localization. They play a major role in photorespiration in photosynthetic tissues while, in germinating seeds, these organelles mediate the catabolism of fatty acids from storage lipid to fuel post-germinative growth (Hu *et al.*, 2012; Bussell *et al.*, 2013), This catabolism is achieved by the β-oxidation pathway, which oxidizes activated fatty acids (acyl-CoA) to acetyl-CoA that may, in turn, enter the TCA cycle, the glyoxylate cycle, and gluconeogenesis (Graham, 2008). *Arabidopsis* mutants of many core β-oxidation proteins are unable to metabolize seed storage lipids and require an exogenous carbon source for seedling establishment. In addition, the plant hormones jasmonic acid (JA) and indole-acetic acid (IAA) are synthesized or matured by β-oxidation. An endogenous precursor to IAA, indole-butyric acid (IBA) and the synthetic auxin 2,4-dichlorobutyric acid (2,4-DB) undergo one cycle of β-oxidation to produce the biologically active auxins IAA and 2,4-dichlorophenoxy-acetic acid (2,4-D) (Baker *et al.*, 2006). Genetic screens that have revealed β-oxidation mutants (Hayashi *et al.*, 1998; Zolman *et al.*, 2000; Eastmond, 2006) have used the sucrose dependence of seedling establishment or the resistance of seedlings to auxin precursors, the latter as assessed by root elongation of mutants on media containing the pro-auxins. Thus, the most readily observable phenotypes of plant β-oxidation mutants are obtained at the seedling stage of the life cycle. By contrast, despite significant impediments to seedling establishment, such β-oxidation mutants usually appear quite normal as mature plants.

In plants, import of all known β-oxidation substrates requires the ABC transporter COMATOSE (CTS), also known as PXA1, PED3, ACN2, and AtABCD1 (reviewed by Verrier *et al.*, 2008). CoA-activated fatty acids are imported by CTS concomitant with CoA cleavage (De Marcos Lousa *et al.*, 2013). Although it remains unknown whether this cleavage occurs on the matrix- or cytosolic side of peroxisomes, fatty acids must be re-activated by Long Chain Acyl-CoA Synthetase (LACS) proteins in peroxisomes in order to enter the β-oxidation cycle (Fulda *et al.*, 2004). β-oxidation then proceeds via four different enzyme activities: (i) acyl-CoA oxidase (ACX), which oxidizes acyl-CoA to 2*E*-enoyl-CoA; (ii) an enoyl-CoA hydratase which oxidizes the 2*E*-enoyl-CoA to 3-hydroxyacyl-CoA, (iii) a 3-hydroxyacyl-CoA dehydrogenase producing 3-oxoacyl-CoA; (iv) 3-ketoacyl-CoA thiolase (KAT) which cleaves an acetyl-CoA molecule from 3-oxoacyl-CoA, leaving the acyl-CoA chain two carbons shorter (Graham, 2008).

Enzymes encoded by small multigene families catalyse each of these steps. In *Arabidopsis thaliana* the ACX family consists of six members that vary in substrate specificity according to acyl-CoA chain-length (Adham *et al.*, 2005). In plants, activities (ii) and (iii) are typically carried out by a single-polypeptide multifunctional enzyme, of which there are two in *A. thaliana* named abnormal inflorescence meristem 1 (AIM1) and multifunctional protein 2 (MFP2) (Richmond and Bleecker, 1999; Rylott *et al.*, 2006). In addition, there are a number of single function hydratases and dehydrogenases that may act on specific substrates, such as auxin precursors (Zolman *et al.*, 2007, 2008; Wiszniewski *et al.*, 2009; Strader *et al.*, 2011). Finally, peroxisomal KAT is encoded by three genes in *A. thaliana* that are known as *KAT1* (At1g04710), *KAT2* (At2g33150), and *KAT5* (At5g48880) (Germain *et al.*, 2001).

*Arabidopsis KAT2* has well-characterized roles throughout plant development. Mutant *kat2* seedlings (also known as *ped1*) do not establish without an exogenous source of sugar and they are resistant to both IBA and 2,4-DB (Hayashi *et al.*, 1998; Germain *et al.*, 2001; Wiszniewski *et al.*, 2009). Three cycles of β-oxidation are required for production of JA from 12-oxo-phytodienoic acid (OPDA), and KAT2 has been shown to contribute to JA production induced by wounding (Cruz Castillo *et al.*, 2004; Afitlhile *et al.*, 2005) and natural senescence (Castillo and Leon, 2008). KAT2 has recently been shown also to contribute significantly to peroxisomal benzoic acid (BA) synthesis (Bussell *et al.*, 2014). By contrast, little is known about the functions of *KAT1* and *KAT5,* although *KAT5* does influence the composition of benzoylated metabolites in seeds (Bussell *et al.*, 2014). Phylogenetic analysis of KAT proteins obtained from sequenced plant genomes showed that KAT2-like and KAT5-like proteins have been conserved in essentially all seed plant lineages, while the *KAT1* gene is a duplication of *KAT2* that is present only in the Brassicaceae family and expressed very weakly in *Arabidopsis* (Wiszniewski *et al.*, 2012). The *KAT5* gene produces two distinct transcripts that encode cytosolic and peroxisomal proteins (Carrie *et al.*, 2007), but this trait also appears to be specific to species in the Brassicaceae family (Wiszniewski *et al.*, 2012).

Amongst *Arabidopsis* β-oxidation mutants *aim1* knockouts are unusual in expressing a strong phenotype in mature plants. Thus, as well as producing seedling roots resistant to pro-auxin, *aim1* mutants have strongly altered leaf and inflorescence development resulting in highly reduced fecundity (Richmond and Bleecker, 1999). CTS and KAT2 have also been shown to be required for full fertility (Footitt et al., 2007a, b), but the mature plants lacking these genes do not exhibit any obvious morphological defects. *KAT2* and *KAT5* genes are expressed strongly in flowers and siliques indicating that thiolases may be functionally important in reproductive tissues (Kamada *et al.*, 2003; Wiszniewski *et al.*, 2012). *KAT2* is co-expressed with genes of β-oxidation, but *KAT5* unexpectedly is co-expressed (Carrie *et al.*, 2007) and co-regulated (Stracke *et al.*, 2007, 2010) with genes of flavonoid biosynthesis.

The dual targeting of KAT5 to peroxisomes and cytosol, the co-expression (and co-regulation) of the gene with those of flavonoid biosynthesis, and the apparent importance of β-oxidation in reproductive tissue suggest undiscovered functions for β-oxidation. The aim of the present work was to investigate KAT5 function, particularly in relation to reproduction and seed germination and to reveal that its function is partially redundant with that of KAT2 in inflorescence development and fertility.

## Materials and methods

### Growth conditions

Surface-sterilized seeds were scattered on 0.5× MS growth media supplemented with 1% (w/v) sucrose, hormones or selective herbicide as required. Seeds were imbibed and stratified for 48h at 4 °C, and grown under continuous light (~100 µE m^–2^ s^–1^) at 20 °C. Seedlings grown for approximately 10–15 d on 0.5× MS growth media were transferred to soil in pots. The soil mix was 2:1 v/v shamrock peat:vermiculite. Rooms were maintained at 22 °C and 60% relative humidity.

### *KAT* mutants

The mutants used in this study are detailed in Table 1. These were obtained from FLAG (Samson *et al.*, 2004) and CSHL (Sundaresan *et al.*, 1995) T-DNA collections. *kat2-1* (Ws-4) was first described in Germain *et al.* (2001), and *kat1* (Ws-4) and *kat5-1* (L*er*) in Wiszniewski *et al.* (2009). *kat2-5* (Ws-4, FLAG_307C02) and *kat5-2* (Ws-4, FLAG_065D06) are new alleles described here. The T-DNA Express primer design server (http://signal.salk.edu/tdnaprimers.2.html) was used for primer design for the newly described lines. RT-PCR primers were designed to flank the insertion sites and used to screen each line for the absence of a full-length transcript as an indicator that they were transcript knockouts. Primer sequences are given in Supplementary Table S1 at *JXB* online. *kat2* and *kat5* mutants were crossed and the F_2_ generation segregated to make double mutants. As *kat2 kat5* double mutants were infertile, they were maintained as sesquimutants (homozygous for one mutation and heterozygous for the other). *kat2-1/kat2-1 kat5-2/KAT5* or *kat2-1/KAT2 kat5-2/kat5-2* were analysed for segregation of *kat2-1* and *kat5-2* alleles. A Chi square test was used to test deviation from the expected segregation ratio (3:1) at each heterozygous locus.

**Table 1.** Thiolase mutant lines used in this study

Gene	Line	Allele	Ecotype	Reference
*KAT1* (At1g04710)	FLAG_589G05	*kat1-1*	Ws-4	Wiszniewski *et al.* (2009)
*KAT2* (At2g33150)	T-DNA	*kat2-1*	Ws-4	Germain *et al.* (2001)
	FLAG_307C02	*kat2-5*	Ws-4	This study
*KAT5* (At5g48880)	CSHL_ET5406	*kat5-1*	L*er*	Wiszniewski *et al.* (2009)
	FLAG_065D06	*kat5-2*	Ws-4	This study

### Seed and seedling phenotypic characterization

Hypocotyl elongation and germination were assayed to test for sucrose-dependence, and response to 2,4-DB was determined as described in Wiszniewski *et al.* (2009). Germination frequency was assayed using seed that had been after-ripened for at least 8 weeks. 250–300 seeds were scattered on water-agar (0.8% w/v) media and immediately placed under illumination with no stratification. Seeds were scored for germination every 24h and the experiment was replicated four times.

### Fatty acid analysis

Seed weight and fatty acid composition were determined using seed harvested from soil-grown plants under long-day conditions. 500–600 seeds from individual plants were counted and weighed. For analysis of fatty acid contents of dry seeds, counted and weighed pools of seed (approximately 10mg) were extracted and measured by GC-MS as described in Wiszniewski *et al.* (2009). A similar protocol was used for analysis of fatty acids in germinating *kat5-2* seedlings except that 50 seedlings were used for analysis.

### Pollen viability

Pollen viability was assessed using the method of Alexander (1969). Pollen was spread on microscope slides and immersed in a drop of stain solution [9.5% (v/v) ethanol, 25% (v/v) glycerol, 2% (v/v) glacial acetic acid, 5% (w/v) phenol, 0.05% (w/v) malachite green, 0.05% (w/v) fuchsin acid, 0.005% (w/v) orange G]. Slides were briefly flamed (without boiling) prior to microscopy.

### Flavonoid staining

Seeds were imbibed for 24h in water, then stained with 0.25% (w/v) diphenylboric acid 2-aminoethyl ester (DPBA) for 15min. The seed coat was removed, and embryos were viewed using an Olympus BX61 epifluorescence microscope with a FITC filter. *tt4-1* seeds (Shirley *et al.*, 1995) were used as a flavonoid-deficient control.

### Plasmid construction and mutant complementation

*KAT5.1* and *KAT5.2* cDNAs, corresponding respectively to cytosolic (KAT5cyt) and peroxisomal (KAT5px) isoforms of the encoded protein, were isolated by RT-PCR (Biorad iScript select) from mature rosette leaf RNA, and ligated into the pCR2.1-TOPO vector (Invitrogen). Cloning primer sequences are given in Supplementary Table S1 at *JXB* online. Following sequencing to confirm error-free DNA replication, *KAT5.1* and *KAT5.2* cDNAs were cloned into pGREEN0180A, a vector based on pGREEN0179 (Hellens *et al.*, 2000), but which had been modified for 35S over-expression and Gateway cloning (Wiszniewski *et al.*, 2009). *Agrobacterium tumefaciens* strain GV3130 was used for *A. thaliana* floral dip (Clough and Bent, 1998) to introduce the constructs into *kat* sesquimutants. Primary transformants were selected using hygromycin resistance of the transgene, and PCR genotyping for the segregating thiolase locus.

### Western blots

Western blotting was done essentially according to Germain *et al.* (2001). Twenty micrograms of soluble protein extracted from 7-d-old seedlings was separated on 12% pre-cast acrylamide gels (Bio-Rad Miniprotean TGX Cat #456–1043) and transferred to Hybond ECL membrane using a Bio-Rad mini-transfer cell. Blots were probed with 1/1000 dilution of KAT2 primary antibody (Germain *et al.*, 2001). The 2° antibody was HRP-conjugated goat anti-rabbit (1/1000; Life Technologies, G21234), which was detected using Bio-Rad Clarity Western ECL substrate (Cat #170–5060). For a loading control, blots were re-probed with a 1/2000 dilution of α-tubulin (Sigma T-5168), for which the 2° antibody was a 1/5000 dilution of alkaline phosphatase goat anti-mouse (Sigma A-2179) and detection used Immune-Star AP (Bio-Rad **#**170–5018). Chemiluminescence was visualized using an ImageQuant RT ECL Imager.

## Results

### *kat1* and *kat5* mutants grow and develop normally

Knockout mutants in the Ws-4 background were available for each of the *KAT* genes, so the available *kat* mutants in this ecotype (Table 1) were obtained including new mutant alleles of *KAT2* and *KAT5* (*kat2-5* and *kat5-2*, respectively), which were determined to be knockouts by the absence of transcripts (see Supplementary Fig. S1 at *JXB* online). Since *kat5-2* is the only mutant in the Ws-4 background, for some experiments where it was desirable to include a second allele of *kat5*, the previously described *kat5-1* (L*er*) was used.

**Fig. 1.**
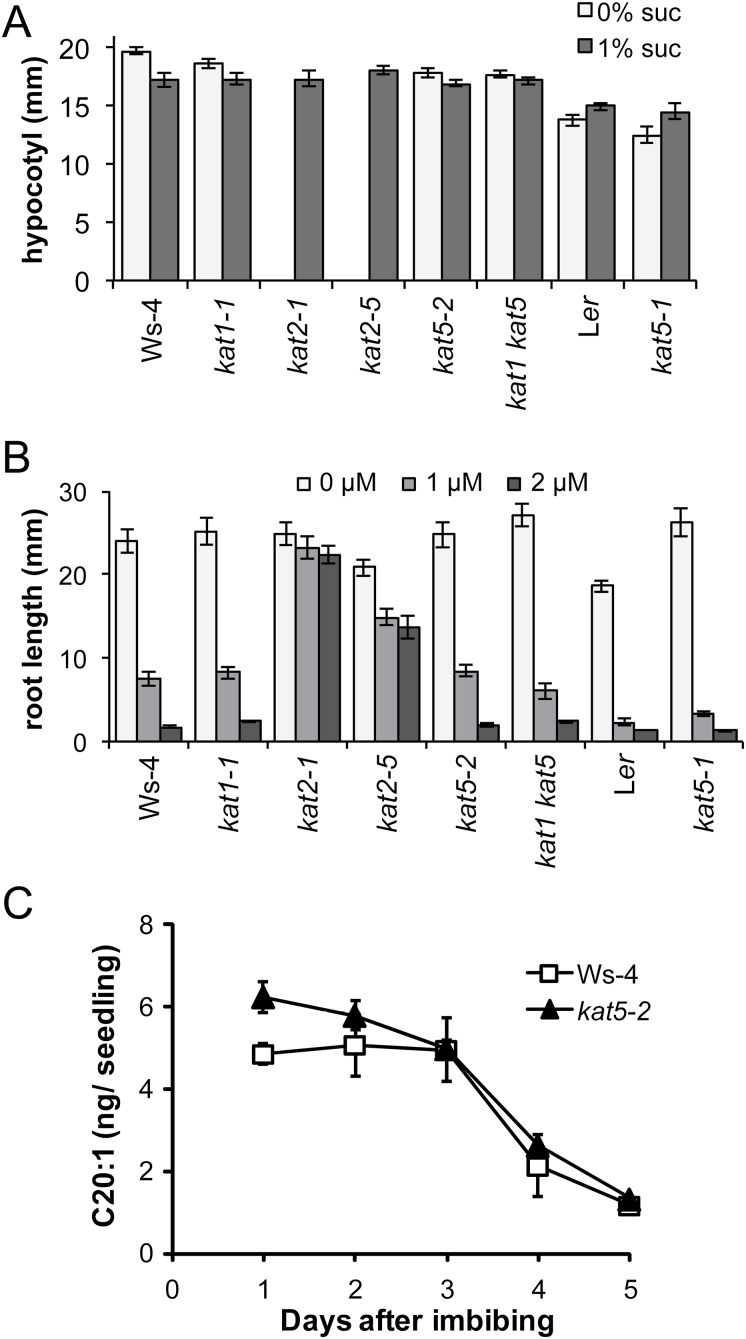
β-oxidation phenotypes of *kat* mutants. (A) Length of etiolated hypocotyls. Plants were grown in the dark for 4 d on 0.5× MS media supplemented with or without 1% (w/v) sucrose (*n* ≥14). (B) Inhibition of root growth in response to varying concentrations of 2,4-DB of thiolase mutants after 8 d growth (*n* ≥13). (C) Degradation of the TAG fatty acid marker C20:1 in Ws-4 and *kat5-2* from 2–5 d post-stratification. Seeds were germinated on plates and 50 seedlings were collected at 24h intervals. In each panel, values represent mean ±SE (*n*=4).

**Fig. 2.**
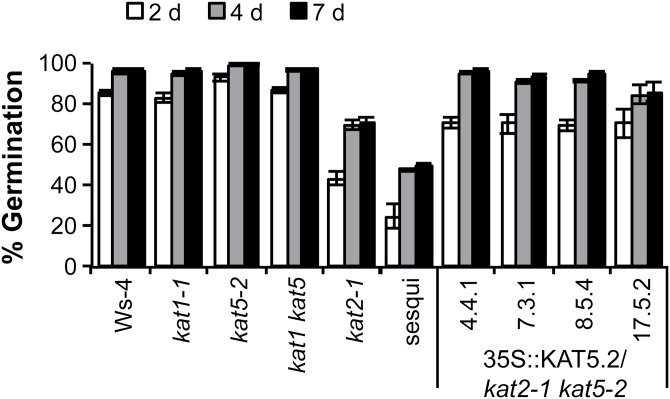
Analysis of germination of *kat* mutants. Germination frequency of seeds of *kat* mutants, a *kat2 kat5* sesquimutant (*kat2-1/kat2-1 kat5-2*/*KAT5*), and a *kat2-1 kat5-2* double homozygous mutant complemented with *35S::KAT5px* (peroxisome-targeted KAT5). Approximately 200 after-ripened, age-matched seeds were scored daily for cumulative % germination on water-agar media. Values are mean ±SE (*n*=4). The *kat2 kat5* double mutant can only be propagated with at least one wild-type KAT allele, and can only be complemented by peroxisome-targeted KAT (see text).

Like other *kat2* mutants (Hayashi *et al.*, 1998; Germain *et al.*, 2001; Wiszniewski *et al.*, 2009), *kat2-5* was dependent on exogenous sugar supply for seedling establishment, was resistant to 2,4-DB (Fig. 1A, B), and exhibited reduced germination capacity (Fig. 2). For these experiments seeds had been after-ripened for 8 weeks and stored at –20 °C to maintain high viability, which overcomes the need to nick the seed coat as reported in some studies (Pinfield-Wells *et al.*, 2005). *kat1-1*, *kat5-2*, and the double mutant *kat1-1 kat5-2* seedlings were indistinguishable from the wild type (Fig. 1A, B). Fatty acid catabolism during the early growth of *kat2-1* seedlings is retarded (Germain *et al.*, 2001; Wiszniewski *et al.*, 2009), but appears to proceed normally in *kat5-2* (Fig. 1C). Thus, as expected, given the severity of *kat2* phenotypes, KAT1 and KAT5 do not apparently play a significant role in oil catabolism or the processing of pro-auxins during seedling establishment.

**Fig. 3.**
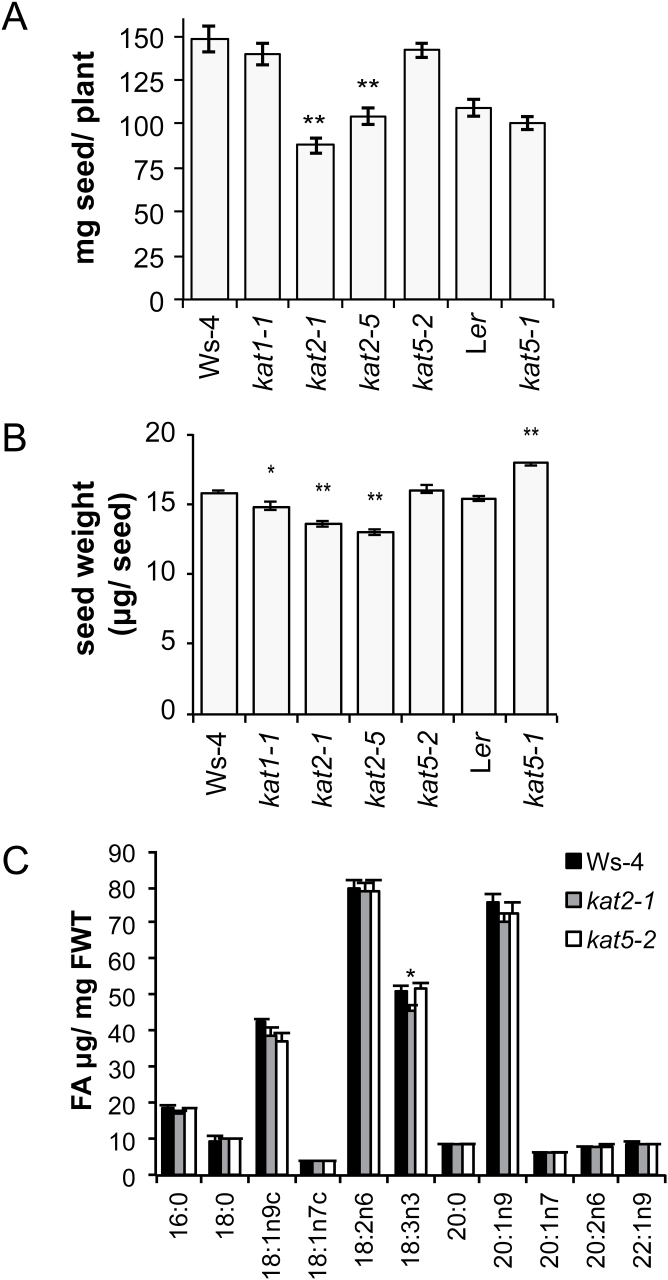
Seed mass and yield-per-plant of *kat* mutants. (A) Seed yield of *kat* mutant plants (*n* ≥7). (B) Mean seed weight of pools of 500–600 *kat* mutant seeds (*n* ≥7). (C) *kat2-1* and *kat5-2* seed oil content of individual fatty acid species normalized for seed weight (*n*=4). (Values are mean ±SE; *P <0.05, **P <0.01, ANOVA).

After seedling establishment, single mutants *kat1-1*, *kat2-1*, *kat2-5*, *kat5-1*, and *kat5-2* appeared quite similar to wild-type plants (see Supplementary Fig. S2 at *JXB* online). Since *KAT2* and *KAT5* are highly expressed in developing seeds (Wiszniewski *et al.*, 2012) and *KAT2* is required for full fertility in *A. thaliana* (Footitt *et al.*, 2007a) the effect of *kat* mutations on seed production was investigated. As reported previously (Footitt *et al.*, 2007a), *kat2* mutants produced lower seed yield per plant and the seeds were individually of lower weight than wild type (Fig. 3). For *kat1-1,* the total seed yield per plant was unaltered but individual seeds weighed slightly less than those of the wild type (Fig. 3A, B). Neither of the *kat5* knockout mutants was altered in total seed mass produced per plant compared with their respective wild types, but the mass of individual *kat5-1* seeds was greater than for L*er* wild-type seeds (Fig. 3A, B). The seed oil content of *kat2-1* and *kat5-2* was quite similar (on a fresh-weight basis) to the wild type for a range of fatty-acid species, the main exception being C18:3n3 (linolenic acid) which was significantly lower in *kat2-1* relative to the wild-type (*P* <0.05; Fig. 3C).

### *kat5* mutants exhibit normal flavonoid-related phenotypes

A role for KAT5 in flavonoid biosynthesis has previously been hypothesized based on co-expression and co-regulation of *KAT5* with genes of that pathway (Carrie *et al.*, 2007; Stracke *et al.*, 2007, 2010) (see also Supplementary Fig. S3A, B at *JXB* online). To investigate a possible functional relationship between KAT5 and enzymes of flavonoid biosynthesis such as chalcone synthase (encoded by the *TRANSPARENT TESTA 4* gene, *TT4*), the seed coat colour of *kat* mutants was compared with that of Ws-4, *tt4*, and L*er* (the background of the *tt4* mutant). All *kat* mutants and the wild type had pigmented seed coats while those of *tt4* were transparent (see Supplementary Fig. S3C at *JXB* online). Seeds were imbibed for 24h, seed coats removed, and seedlings stained with diphenylboric acid 2-aminoethyl ester (DPBA), which fluoresces specifically in the presence of flavonoids (Peer *et al.*, 2001). Fluorescence was clearly observed in the wild type and in *kat2* and *kat5* mutants, but was absent in *tt4* (see Supplementary Fig. S3D at *JXB* online), indicating that the gross alteration in flavonoid content seen in flavonoid mutants was not apparent in *kat5*.

### Analysis of *kat* double mutants

Three double mutant combinations were examined: *kat1 kat2*, *kat1 kat5*, and *kat2 kat5*. The *kat1-1 kat5-2* double mutant was fully fertile and indistinguishable from the wild type in seed germination (Fig. 2) and seedling growth (Fig. 1) and so was not studied further. By contrast, many hundreds of F_2_ and F_3_ plants, generated by crossing *kat1-1* with either *kat2-1* or *kat2-5*, were screened but we were unable to obtain either sesquimutant (plants homozygous at one locus but heterozygous for the other) or double homozygote plants. It is concluded from this that *KAT1*, although barely expressed (Wiszniewski *et al.*, 2012), may be partially redundant with *KAT2* for some essential function and that the loss of both genes is lethal. Alternatively, the *kat1-1* mutant carries a closely linked secondary mutation that is incompatible with *kat2*. However, alternative alleles of *kat1* were not available to explore this further.

Crosses to generate the *kat2 kat5* double mutant readily yielded fertile sesquimutant plants. Progeny from *kat2-1 kat5-2* sesquimutant parents that were segregating at either the *KAT2* or the *KAT5* locus were analysed by PCR analysis of extracted DNA to determine the genotype at the heterozygous locus (Table 2). Seed for this experiment were cold-stratified and grown on MS medium containing sucrose to maximize germination and growth. The progeny of individual *kat2-1/kat2-1 kat5-2/KAT5* plants did not differ significantly from the expected ratio of 1:2:1 for segregation at a single recessive locus (i.e. for *KAT5*/*KAT5*:*kat5-2*/*KAT5*:*kat5-2*/*kat5-2*). However, segregation analysis of *kat2-1/KAT2-1 kat5-2/kat5-2* plants yielded a ratio at the *KAT2* locus of about 4:4:1, significantly different from 1:2:1 (*P* <0.01) and under-represented in both homozygotes (*kat2-1/kat2-1*) and heterozygotes (*kat2-1/KAT2-1*).

**Table 2.** Segregation analysis of *kat2-1 kat5-2* mutants homozygous at one locus but segregating at the otherSegregating mutant seedlings were genotyped by PCR to determine their segregation ratio, and this was compared with the expected 1:2:1 ratio for a single recessive mutation by χ^2^ analysis. The frequency (%) of each genotype is given in brackets.

Media	Background mutation	Segregating mutation			Total	χ^2^ (1:2:1 hypothesis)
Wild-type	Heterozygous	Homozygous
0.5× MS+1% suc	*kat5-2*	*KAT2*/*KAT2*	*kat2-1*/*KAT2*	*kat2-1*/*kat2-1*		
		42 (46%)	40 (44%)	9 (9.9%)	91	3.3×10^–6^
0.5× MS+1% suc	*kat2-1*	*KAT5*/*KAT5*	*kat5-2*/*KAT5*	*kat5-2*/*kat5-2*		
		28 (30%)	46 (49%)	19 (20%)	93	0.42
Water–agar	*kat2-1*	*KAT5*/*KAT5*	*kat5-2*/*KAT5*	*kat5-2*/*kat5-2*		
		50 (28%)	105 (58%)	26 (14%)	181	0.0041

The data presented in Table 2 may be regarded as arising from maximum germination potential, due to the stratification of seeds and their incubation on medium containing both ${\text{NO}}_{3}^{–}$ and sucrose, known germination promoters. To assess further the effect of *kat2-1* and *kat5-2* single and sesquimutants on germination potential, seed batches from plants that had been grown at the same time and under the same conditions were sown on water-agar and placed directly in the light without stratification. By 4 d, Ws-4 and *kat5-2* had germination frequencies greater than 95% (Fig. 2). By contrast, germination at 7 d was 70% for *kat2-1* (homozygous seed), and only 50% for *kat2-1*/*kat2-1 kat5-2*/*KAT5* (note that single parent sesquimutant seed pools showed approximately 1:2:1 segregation ratio at the *KAT5* locus, Table 2). It is inferred that the reduction in germination efficiency in the sesquimutant seed pools (Fig. 2) was due to double homozygotes among the progeny. Indeed, genotyping of seeds that had germinated after 7 d on water-agar indicated a deficiency of *kat5-2* homozygotes such that the ratio of KAT5/KAT5:KAT5/*kat5-2*:*kat5-2*/*kat5-2* was approximately 2:4:1, significantly different (*P* <0.01) from the expected 1:2:1 (Table 2). This implies that *kat2-1 kat5-2* double homozygote seeds are less likely to germinate on water-agar than wild-type or sesquimutant seeds.

### Double mutants of *kat2* and *kat5* recapitulate the *aim1* phenotype

**Fig. 4.**
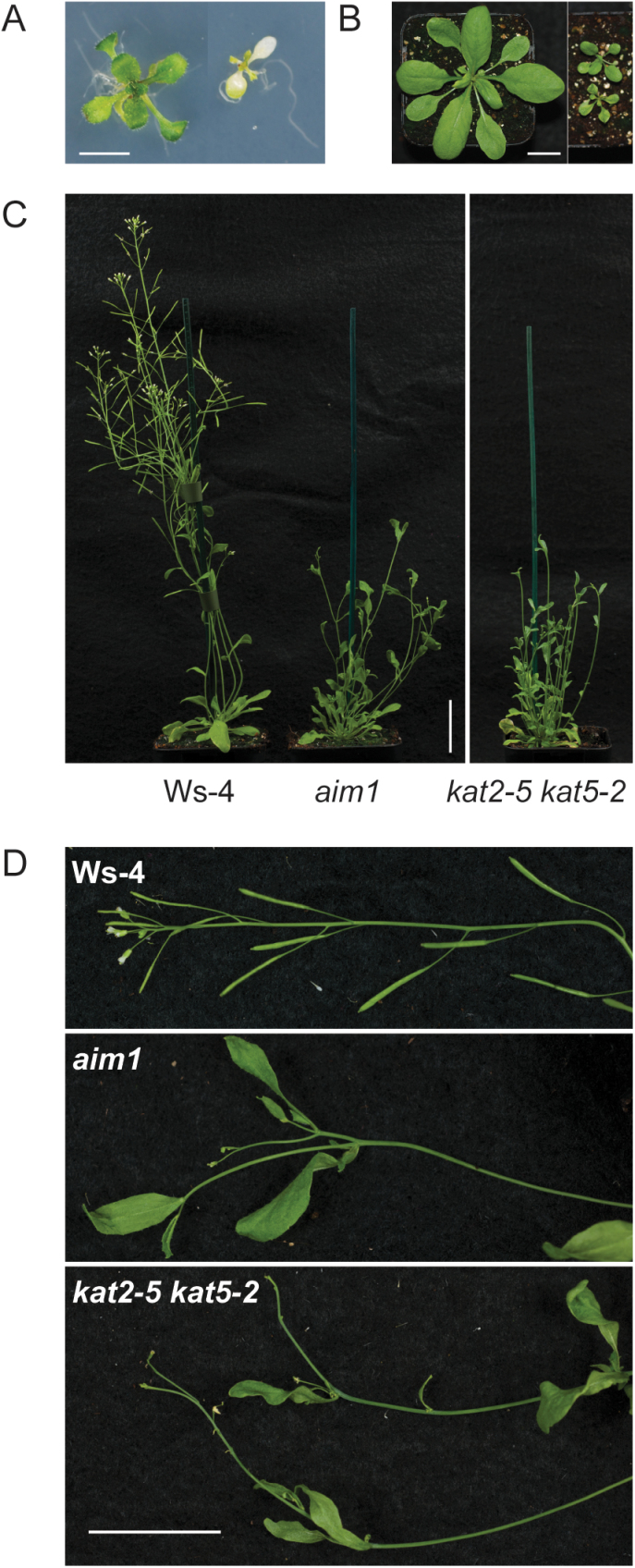
*kat2 kat5* double mutant phenotypes at various stages of plant development. (A) Ws-4 wild type (left) and *kat2-1 kat5-2* double mutant (right) grown under continuous light for 14 d on 0.5× MS supplemented with 1% sucrose (scale bar, 10mm). (B) Phenotype of such plants grown for a further 22 d after transfer to soil and long days (16/8h light/dark; scale bar, 15mm). (C) Phenotype of 40-d-old Ws-4, 40-d-old *aim1*, and 55-d-old *kat2-5 kat5-2* (scale bar, 30mm). Soil-grown plants were grown initially for 7 d on media containing sucrose to enable the establishment of double knockouts which were confirmed by PCR genotyping. (D) Close-up images of comparable lengths of typical inflorescences of the plants depicted in (C) which highlights the malformation and ectopic positioning of flowers, siliques, and cauline leaves in inflorescences of *aim1* and *kat2-5 kat5-2* mutants (scale bar, 30mm).

**Fig. 5.**
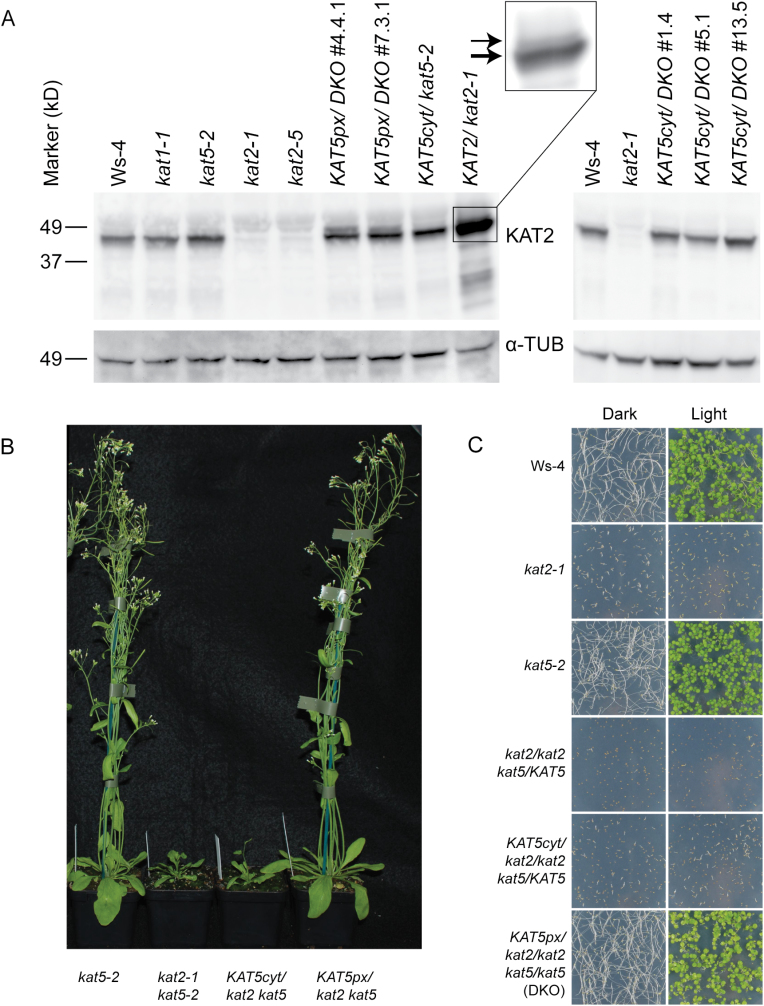
Peroxisomal KAT5 complements *kat2 kat5* double knockouts. (A) Western blot of *kat* mutants. A KAT2 antibody (Germain *et al.*, 2001) was used to probe protein extracted from 7-d-old whole seedlings that had been germinated on 0.5× MS media supplemented with 1% sucrose (left panel) or 2,4-DB (right panel, to select for double knockouts). α-TUB=α-tubulin loading control. The inset depicts resolution of a second, larger band originating in the KAT2 over-expressing line (see text). (B) Restoration of fertility in *kat2 kat5* double knockouts by peroxisomal KAT5 (KAT5px). *kat2 kat5* phenotypes were rescued by constitutive expression (by CAMV 35S promoter) of KAT5px, but not by a variant of the protein targeted to the cytosol (KAT5cyt). Plants were grown in long-day conditions for 35 d. (C) Complementation of the sucrose-dependent phenotype of *kat2 kat5* double knockout. Seedlings were grown on 0.5× MS without sucrose supplement in the dark or in the light for 8 d. *kat2-1/kat2-1 kat5-2*/*KAT5* sesquimutants were unable to establish on media lacking sucrose. The introduction of constitutively expressed KAT5px, but not KAT5cyt, allowed both the recovery of *kat2 kat5* double knockouts and their establishment in the absence of sucrose. Scale bar, 20mm. For all panels, the *kat2 kat5* mutant (DKO) was *kat2-1 kat5-2*.

Homozygous *kat2 kat5* (Ws-4) double knockout seedlings that had been identified by PCR genotyping were grown in soil under long-day conditions for further analysis. They exhibited severe growth defects (Fig. 4). These double mutant seedlings were paler green than the wild type (Fig. 4A). Throughout development, they were slower growing than the wild type or *kat* single mutants, displaying reduced rosette size and delayed flowering (Fig. 4B, C). Mature *kat2 kat5* double knockout plants had reduced apical dominance and shorter inflorescences with atypical internode spacing and altered anatomy. These phenotypes are strongly reminiscent of the *aim1-1* mutant (Richmond and Bleecker, 1999) (Fig. 4B, C). The *kat2 kat5* double mutant was replicated using alternative alleles for each locus (ie. *kat2-1 kat5-2*, *kat2-5 kat5-2*, and *kat2-1 kat5-1)* and obtained similar phenotypes in each case (Figs 4, 5). As there was only one allele for *kat5* in the Ws-4 background, the *kat5* allele in the *kat2-1 kat5-1* double mutant combination was in the L*er* ecotype (Table 1), but this gave results consistent with crosses between Ws-4 mutants. Although *kat2 kat5* mutants were slower growing than *aim1-1* (also in the Ws-4 background), their flowers were similarly malformed, often exhibiting ectopic organ development (Fig. 4D).

At later stages of the life cycle, *kat2 kat5* flowers developed more normally, appearing almost like the wild type. These late developing flowers produced a small amount of pollen, but the siliques of double mutant plants were always empty, even after manual self-pollination. Reciprocal crosses of *kat2-1 kat5-2* and wild-type plants were conducted and wild-type pollen was unable to fertilize *kat2-1 kat5-2* plants. However, Alexander staining showed that pollen from double mutants was viable (see Supplementary Fig. S4 at *JXB* online), and it was able to fertilize wild-type ovules, with such crosses producing heterozygous seed. Exogenous supply of JA has previously been show to restore fertility in the *acx1 acx5* double mutant (Schilmiller *et al.*, 2007), but treatment (as described in that work) with JA was unable to restore fertility to *kat2-1 kat5-2* flowers despite several attempts. Thus there appears to be a defect in gynaecium function in *kat2 kat5* mutants.

### Peroxisomal rather than cytosolic KAT5 is essential

To determine if cytosolic or peroxisomal KAT5 activity could rescue these phenotypes, two transgenes were created, *35S::KAT5.1* and *35S::KAT5.2*. These respectively encoded cytosolic (KAT5cyt) and peroxisomal (KAT5px) variants of KAT5 and were introduced into *kat2-1 kat5-2* sesquimutant parent plants. PCR-genotyping of *KAT2* and *KAT5* loci in plants harbouring these transgenes revealed that KAT5px complemented the *kat2 kat5* double mutants while KAT5cyt did not (Fig. 5). Expression of *35S::KAT5.2* in *kat2-1 kat5-2* was detected by a KAT2 antibody that is reported to require a 5-fold greater amount of KAT5 than KAT2 protein to produce an equivalent signals (Germain *et al.*, 2001) (Fig. 5A). Thus, although the band intensity is comparable to that seen for KAT2 in the wild type (or in the *kat5-2* single mutant), it is likely that the *35S::KAT5.2* drives significant over-expression of KAT5. The mature KAT2 protein (after cleavage of the PTS2 targeting sequence) is expected to be 44.8kDa. A second band of approximately 49kDa was resolved in the *35S::KAT5.2* lines and this corresponds to the size predicted for the unprocessed peptide. The *35S::KAT2*/ *kat2-1* line described in Germain *et al.* (2001) over-expresses KAT2 and also had a heavier band that might correspond to excess unprocessed protein (Fig. 5A, inset). A protein corresponding to the expected size of the KAT5cyt protein is 43.2kDa can be seen in Western blots of confirmed *kat2-1 kat5-2* double knockout plants transformed with *35S::KAT5.1* (Fig. 5A).

Wild-type-like plant habit and fertility was restored only in *kat2-1 kat5-2* plants transformed with *35S::KAT5.2* (Fig. 5B). These results confirm that the infertility phenotype was due to the disruption of peroxisomal thiolase function. The *35S::KAT5.2* transgene also rescued seedling establishment in the absence of exogenous sucrose under both dark and light conditions, while the *35S::KAT5.1* transgene introduced in the *kat2-1*/*kat2-1 kat5-2*/*KAT5* sesquimutant background did not (Fig. 5C). To determine if reduced germinability in sesquimutants was due to the absence of KAT5px (rather than of KAT5cyt), the germination frequency of *kat2-1 kat5-2* double mutants complemented with *35S::KAT5.2* was assayed. Although germination was slightly delayed, expression of KAT5px in multiple independent lines restored germination frequency to almost 100% for double mutants (Fig. 2). Collectively, these data suggest that, while KAT2 is the major thiolase during germination, peroxisomal KAT5 also contributes to germination potential, development of reproductive tissue, and full fertility in *A. thaliana*.

## Discussion

### Functional analysis of *KAT* genes during development

Mutants have been studied to probe the functions of *KAT* genes during plant growth and development. Knockouts of *KAT1* and *KAT5* (and a *kat1 kat5* double knockout) showed no obvious abnormal phenotypes during the life cycle. No evidence was found for a requirement for KAT5 in flavonoid synthesis in seeds, despite co-expression and co-regulation of *KAT5* with genes of that pathway (Carrie *et al.*, 2007; Stracke *et al.*, 2007, 2010). *KAT2* has previously been well characterized and is the primary isoform acting in β-oxidation of fatty acids during seed germination, and also in the processing of hormone precursors including IBA, 2,4-DB, and JA. Accordingly, *kat2* seedlings require exogenous sucrose for establishment, are impaired in the breakdown of TAG and fatty acids, and are resistant to pro-auxins (Hayashi *et al.*, 1998; Germain *et al.*, 2001; Wiszniewski *et al.*, 2009). It was also confirmed that the *kat2* mutant produced fewer and smaller seeds than the wild type (or *kat1* and *kat5* plants). It was not possible to obtain *kat1 kat2* mutants, and *kat2 kat5* double knockout plants were infertile with gross morphological defects to vegetative and reproductive organs.

A role for β-oxidation in seed germination independent of oil breakdown has previously been described for other β-oxidation mutants (Russell *et al.*, 2000; Pracharoenwattana *et al.*, 2005; Pinfield-Wells *et al.*, 2005). For example, it was recently shown that OPDA inhibits germination by acting synergistically with ABA to increase the levels of ABI5 protein (Dave *et al.*, 2011). In β-oxidation mutants, including *cts* and *kat2*, the accumulation of OPDA (that is not converted to JA during seed development) consequently results in reduced germination frequency. *kat2* seed is thus less likely to germinate than the wild type and our new results show that if *kat2* homozygous seed also lacks at least one wild-type *KAT5* allele, fewer seeds germinate and germination is slower.

β-oxidation enzymes are present in the endosperm and embryo during seed development (e.g. see http://bar.utoronto.ca/) and it has been observed that β-oxidation is active in the turnover of lipids in developing embryos (Arai *et al.*, 2002; Chia *et al.*, 2005). Indeed, gluconeogenesis appears to be disrupted in *kat2-1* ovules and they accordingly exhibited reduced respiration rates that may impact embryo development (Footitt *et al.*, 2007a). Alternatively, the accumulation of free-fatty acids may damage embryo cell membranes similar to the observations of damage in leaves of dark-treated *kat2* and *cts* plants (Kunz *et al.*, 2009). It remains unknown whether seed development in these mutants requires β-oxidation in the embryo, embryo sac, endosperm, ovule, ovary or a combination of these tissues. Indeed, seeds of *kat2* are smaller than those of the wild type, *kat1*, or *kat5* (Fig. 3; Footitt *et al.*, 2007a) supporting the notion that β-oxidation plays a role in seed filling or maturation. Reduced individual seed mass has previously been reported in *acx1-1*, *acx1-1 acx2-1*, and *cts-2* mutants (Pinfield-Wells *et al.*, 2005) and in *pex5* mutants (Khan and Zolman, 2010). The *kat2* mutant also produced fewer seeds per plant than the wild type, probably as a result of the abortion of some seeds in each silique (Footitt *et al.*, 2007a). Extremely compromised fertility is seen in some other β-oxidation mutants, including embryo lethality of *aim1 mfp2* and *acx3 acx4* double mutants (Rylott *et al.*, 2003, 2006). It is interesting to note, however, that these reports are for mutants in the Ws-4 background and that a Col-0 *acx3 acx4* double knockout mutant is fertile, pointing to ecotype-specific effects of altered β-oxidation capacity in *Arabidopsis* (Khan *et al.*, 2012). While the present study has investigated the *aim1* and *kat2 kat5* double mutant phenotypes in the Ws-4 ecotype, equivalent studies of these genes in other *Arabidopsis* ecotypes would be valuable but have not yet been reported.

The *kat2 kat5* double mutant is infertile, producing no seeds, even when treated with wild-type pollen. The *KAT1* gene alone is thus insufficient for, or incapable of, conferring fertility. As *kat2 kat5* can produce viable pollen, and fertility is not restored by the application of JA, its infertility may be due to compromised megagametophyte development or to an inability of the mutant style to support or direct fertilization. The latter has been reported for the *abstinence by mutual consent* (*amc*) mutant, an allele of *pex13*. However, *amc* can only exist as a heterozygote: homozygotes cannot be generated at all from heterozygous parent plants (Boisson-Dernier *et al.*, 2008). Homozygous *kat2 kat5* embryos are capable of developing into viable seeds if the parent plant contains one wild-type allele of either *KAT2* or *KAT5*. Moreover, fertility (and germinability) was restored to *kat2 kat5* double mutants by constitutive expression of peroxisome-targeted KAT5. This indicates that *KAT2* and *KAT5* genes are at least partially redundant in determining fertility and that the inability of native KAT5 to compensate for the loss of KAT2 in *kat2* mutants may be due to a sub-threshold amount of KAT activity or to inappropriate timing and spatial expression patterns of *KAT5*. Peroxisomal KAT may supply essential metabolites to, or remove inhibitory metabolites from, the ovule sac or embryo. Alternatively, KAT activity may be required for proper gynaecium and ovule development, rather than play a direct role in the processes of fertilization, embryogenesis, and seed development.

### β-oxidation is essential for normal inflorescence development and plant fertility

*kat2 kat5* double mutants had slow growth, but exhibited proliferation of abnormal inflorescences including ectopic positioning of reproductive organs. The growth and development of *kat2 kat5* plants was similar to that of the *aim1* mutant. The possibility was considered that this may be due to impaired IAA synthesis via β-oxidation of IBA, since disruption to auxin metabolism can affect shoot branching (Bennett *et al.*, 2006). However, this is not supported by an *ech2 ibr1 ibr3 ibr10* quadruple mutant, which has severe defects in the conversion of IBA to IAA in peroxisomes. The quadruple mutant exhibits smaller cotyledons, slower leaf development, and delayed flowering, but it has no gross morphological defects at maturity and its inflorescences are fertile and appear similar to those of wild-type plants (Strader *et al.*, 2011). The pale green leaves of *kat2 kat5* plants were reminiscent of the reduced chlorophyll observed in the *pex5-10* mutant that lacks a full-length PEX5 protein (Khan and Zolman, 2010). *pex5-10* grew relatively normally after seedling establishment and was more similar to *kat2* single mutants in that it exhibited poor germination and reduced weight of individual seeds, but the phenotype of mature plants did not resemble *aim1*.

By contrast, the *ped1 ped3* double mutant combination (which is in the L*er* background and is allelic to *kat2 cts*) had wavy irregular leaves and dwarfed, abnormal inflorescences (Hayashi *et al.*, 2002) and appears to be very similar to *aim1*. An extreme case of impaired shoot growth of a β-oxidation mutant is the citrate synthase double mutant *csy2 csy3* in which the accumulation of peroxisomal acetyl-CoA (rather than he absence of a particular product of β-oxidation) may explain the phenotype (Pracharoenwattana *et al.*, 2005). Similarly, the shoot developmental abnormalities of *kat2 kat5* (and/or *aim1*) could be due to the accumulation of a β-oxidation precursor or intermediate rather than due to the absence of a specific product such as IAA or JA. Given that, after seedling establishment, *cts* mutants exhibit normal shoot and inflorescence development, it seems unlikely that the build-up of extra-peroxisomal precursors is responsible for the altered inflorescence development. Alternatively, CTS-independent routes for the import of substrates such as the JA-precursor OPDA have been proposed (Theodoulou *et al.*, 2005) and, if this is possible for other substrates, loss of CTS alone may be insufficient to produce severe phenotypes.

To test the possibility that intra-peroxisomal accumulation of a β-oxidation intermediate might explain these phenotypes, an attempt was made to make a *cts aim1* double mutant. *AIM1* and *CTS* genes are both located on chromosome 4 (AGIs At4g29010 and At4g39850, respectively). Despite readily deriving a crossover between these loci, and then screening in excess of 150 F_3_ plants, we were unable to generate double mutants, suggesting that this mutant combination is lethal. It is proposed that *aim1*-like phenotypes result from a blockage to β-oxidation caused by severe reduction of an essential metabolic capacity (e.g. *aim1*, *or kat2 kat5*) or that reduced metabolic capacity in combination with a loss of the primary peroxisome substrate import capability in *ped1 ped3* (*kat2 cts*) or *cts aim1*, precludes the production of an essential metabolite or results in the accumulation of an intermediate that is toxic to plant development. The future identification of such intermediates may reveal a new metabolic function for peroxisomes.

## Supplementary data

Supplementary data can be found at *JXB* online.

Supplementary Fig. S1. Identification of new KAT mutants.

Supplementary Fig. S2. Appearance of mature wild type and *kat* single mutants.

Supplementary Fig. S3. KAT5 is not required for flavonoid biosynthesis.

Supplementary Fig. S4. Viability of *kat2 kat5* double mutant pollen

Supplementary Table S1. Oligonucleotide sequences.

Supplementary Data
