# Supplementary material for: Knockout of the two evolutionarily conserved peroxisomal 3-ketoacyl-CoA thiolases in Arabidopsis recapitulates the abnormal inflorescence meristem 1 phenotype
Source: J Exp Bot. 2014 Oct 7;65(22):6723–33. doi: 10.1093/jxb/eru397 (PMC4246196; doi:10.1093/jxb/eru397)
Supplement: Supplementary Data [file supp_65_22_6723__index.html]

Knockout of the two evolutionarily-conserved peroxisomal 3-ketoacyl-CoA thiolases in Arabidopsis recapitulates the abnormal inflorescence meristem 1 phenotype — Knockout of the two evolutionarily conserved peroxisomal 3-ketoacyl-CoA thiolases in Arabidopsis recapitulates the abnormal inflorescence meristem 1 phenotype — Supplementary Data 

# Knockout of the two evolutionarily conserved peroxisomal 3-ketoacyl-CoA thiolases in *Arabidopsis* recapitulates the *abnormal inflorescence meristem 1* phenotype

## Supplementary Data

Data files

**Files in this Data Supplement:**

- Supplementary Data - Supplementary Data
- Supplementary Data - Supplementary Data
