# Supplementary material for: Knockout of the two evolutionarily conserved peroxisomal 3-ketoacyl-CoA thiolases in Arabidopsis recapitulates the abnormal inflorescence meristem 1 phenotype
Source: J Exp Bot. 2014 Oct 7;65(22):6723–33. doi: 10.1093/jxb/eru397 (PMC4246196; doi:10.1093/jxb/eru397)
Supplement: Supplementary Data [file supp_eru397_Sup_Figs_1_4.pdf]

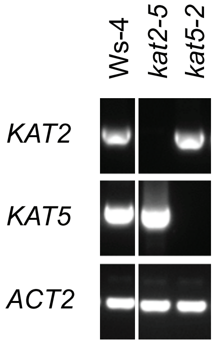

Figure S1. Identification of new KAT mutants. *kat2-5* (FLAG_307C02) was genotyped using the primers FLAG_307C02_LP and FLAG_307C02_RP in conjunction with the border primer FLAG_LB4. Absence of full-length transcript was also confirmed by RT-PCR with the FLAG_307C02 primer pair. *kat5-2* (FLAG_065D06) was genotyped using the primers FLAG_065D06_LP and FLAG_065D06_RP in conjunction with the border primer FLAG_LB4. RT-PCR using primers KAT5-RT-LP and KAT5-RT-RP confirmed the absence of full-length transcript from *kat5-2*. The gel depicts the RT-PCR of *KAT2* and *KAT5* transcripts in Ws-4 (wild-type), *kat2-5* and *kat5-2* mutants. Primer sequences are given in Table S1.

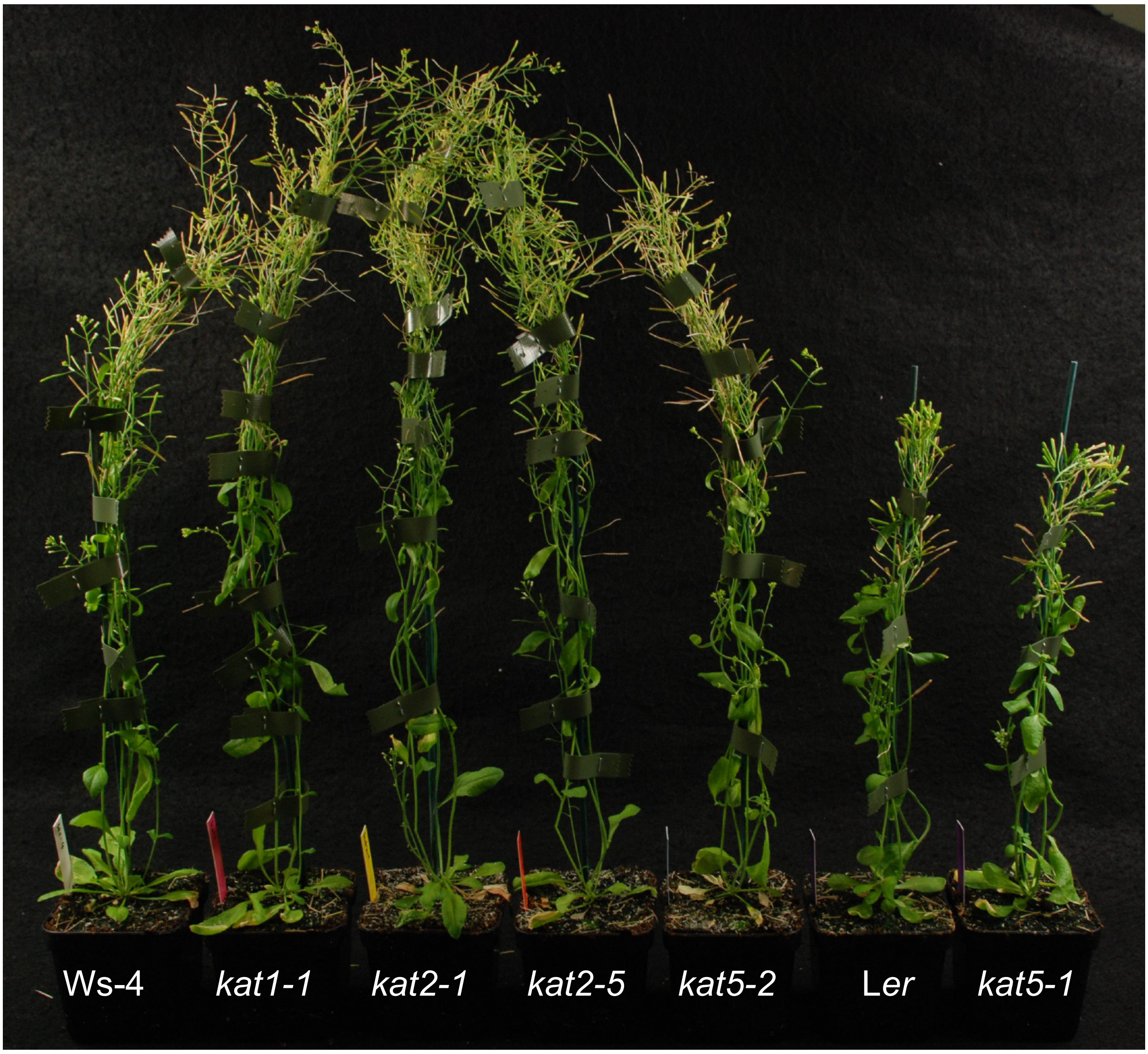

Figure S2. Appearance of mature wild type and *kat* single mutants. Plants were grown for 10 d in continuous light on sucrose-containing media followed by 46 d in soil under long day conditions. Aside from the dependence of the *kat2* mutant on sucrose for seedling establishment and their slightly lower seed yield, there were no discernable differences in gross phenotype of the single mutants compared to their respective wild types.

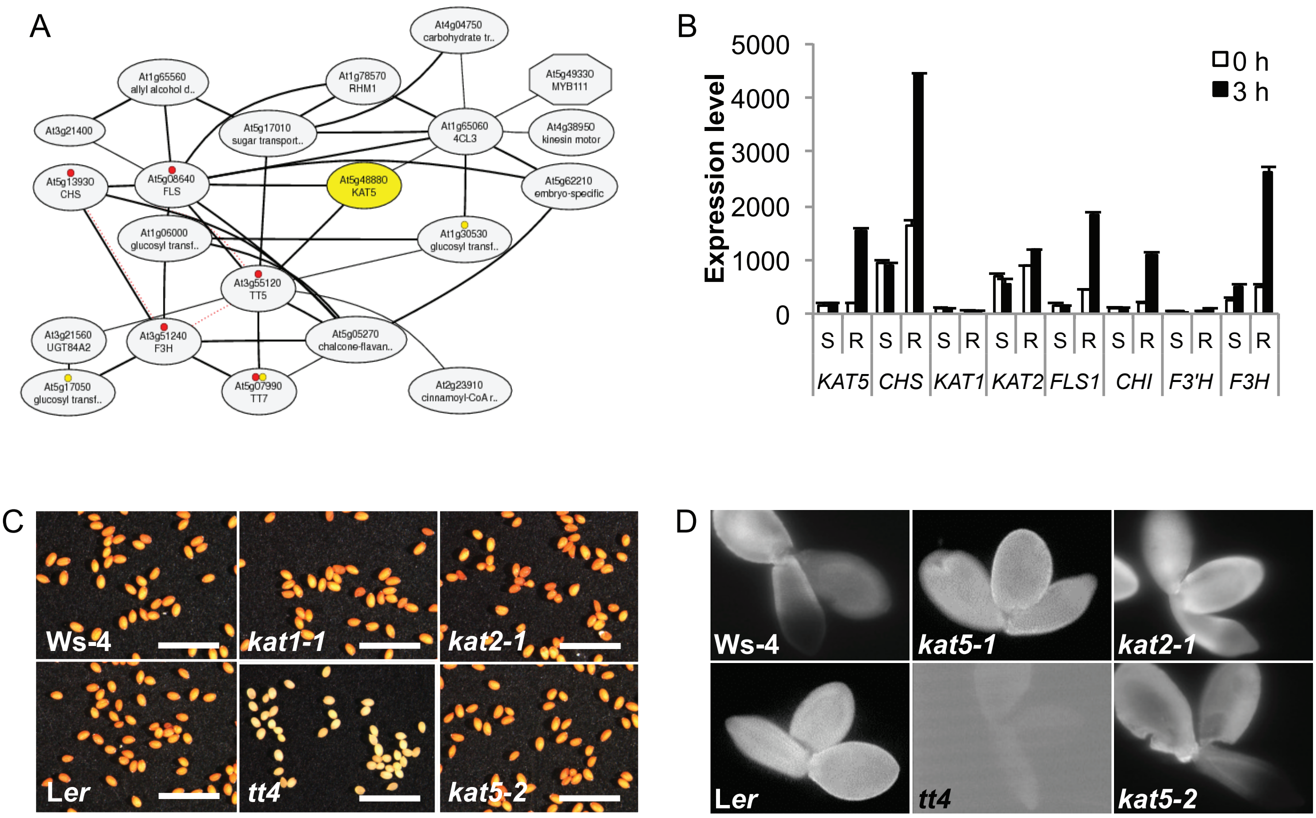

Figure S3. KAT5 is not required for flavonoid biosynthesis. (A) *KAT5* is coexpressed with flavonoid biosynthesis genes. Analysis of co-expression using ATTEDII (http://atted.jp/) shows that *KAT5* clusters within a network of genes related to flavonoid biosynthesis. Yellow and red dots refer to biosynthesis of secondary metabolites (KEGG ID: ath01110) and flavonoid biosynthesis (KEGG ID: ath00941) respectively. (B) KAT5 and flavonoid gene expression is up-regulated by UV-B irradiation. Data retrieved from the BAR Arabidopsis EFP Browser (http://bar.utoronto.ca/) showed up-regulation of *KAT5* and flavonoid biosynthesis genes in root, but not shoot tissue 3 h after treatment with UV-B irradiation. (C, D) *kat5* mutant seeds have no obvious flavonoid-related phenotypes, as evidenced by (C) the appearance of imbibed seeds of *kat* mutants compared to the flavonoid deficient *tt4* mutant (Scale bar = 2 mm), and (D) the evidence of DPBA fluorescence in 24 h imbibed seeds. In (D), DPBA fluoresces in the presence of flavonoids. The testa was removed prior to DPBA-staining, and high gain applied to the *tt4* panel to demonstrate the absence of any staining.

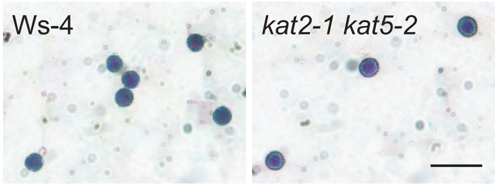

Figure S4. Viability of *kat2 kat5* double mutant pollen. Ws-4 and *kat2-1 kat5-2* pollen was stained with Alexander solution, and plants were shown to be capable of producing viable pollen. Pollen has purple staining in the cytoplasm indicating viability. The outer exine layer stains green. Scale bar, 50 µm.
